# Craniofacial Analysis of Lateral Cephalograms in Obstructive Sleep Apnea—An Exploratory Case–Control Study

**DOI:** 10.3390/diagnostics16081130

**Published:** 2026-04-09

**Authors:** Janine Sambale, Janine Hass, Ulrich Koehler, Heike Maria Korbmacher-Steiner

**Affiliations:** 1 Department of Orthodontics, Clinic of Dentistry, Marburg University, 35039 Marburg, Germanykorbmacher@staff.uni-marburg.de (H.M.K.-S.); 2 Interdisciplinary Center of Sleep Medicine, Department of Pneumology, Marburg University, 35043 Marburg, Germany

**Keywords:** obstructive sleep apnea, lateral cephalogram, posterior airway space, soft palate, hyoid

## Abstract

**Background**: The clinical value of lateral cephalograms for obstructive sleep apnea (OSA) risk assessment remains controversial, largely because previous case–control studies often lacked objective exclusion of OSA in control subjects and insufficiently controlled for confounding. This age-matched case–control study evaluated whether craniofacial characteristics differ between individuals with and without OSA and whether these craniofacial measurements independently predict OSA-related outcomes after adjustment for relevant confounders. **Methods**: A total of 54 adults were included (27 with OSA and 27 without OSA). OSA was defined by poly(somno)graphy (apnea–hypopnea index [AHI] ≥ 5). Control subjects were prospectively recruited, and OSA was excluded through polygraphy (AHI < 5). Lateral cephalograms were used to assess six PAS levels (P1–P6), 16 hyoid- and soft palate-related parameters, and sagittal/vertical skeletal characteristics. Potential confounders were controlled for by adjustment for BMI and craniofacial skeletal pattern. The PAS measurements were defined as the primary endpoint; soft palate and hyoid-related variables were considered secondary exploratory endpoints. Statistical analyses included independent samples *t*-tests, multiple linear regression models, and sensitivity analyses adjusted for sex. **Results**: Craniofacial skeletal characteristics did not differ between groups. PAS dimensions showed no significant intergroup differences and were not independently associated with AHI after adjustment, whereas BMI consistently emerged as the strongest predictor. Uvula length and thickness were significantly greater in the OSA group; however, neither parameter independently predicted AHI in regression models. In contrast, subjects with OSA exhibited a significantly more inferior/anterior hyoid position across multiple models. In the primary regression models, several hyoid-related variables were associated with AHI. However, these associations were attenuated in additional sensitivity analyses after adjustment for sex and were no longer consistently statistically significant. Sex was a relevant covariate in several models. **Conclusions**: Static PAS measurements derived from lateral cephalograms provide no clinically meaningful information for OSA screening or risk stratification. Although several hyoid-related variables were associated with AHI in primary models, these associations were attenuated after adjustment for sex and should therefore be interpreted as exploratory. When lateral cephalograms are already clinically indicated, hyoid position may provide complementary anatomical information, but its independent predictive value remains uncertain.

## 1. Introduction

Obstructive sleep apnea (OSA) is a common and often underdiagnosed sleep-related breathing disorder characterized by recurrent episodes of partial or complete upper airway obstruction during sleep, resulting in intermittent hypoxemia, cortical arousals, and sleep fragmentation. It has an estimated global prevalence of nearly 1 billion individuals, and it is associated with cardiovascular disease, metabolic disorders, neurocognitive impairment, stroke, and excessive daytime sleepiness [1,2,3,4]. As craniofacial morphology influences upper airway anatomy, OSA has become an increasingly relevant condition within orthodontic and surgical dentofacial orthopedic practice.

The pathophysiology of OSA reflects a complex interaction between anatomical susceptibility and neuromuscular control of the upper airway [5]. Craniofacial skeletal pattern, mandibular position, and vertical facial morphology may affect pharyngeal airway size and collapsibility, commonly described in individuals with skeletal class II or increased vertical dimensions [6,7]. In addition, chronic intermittent hypoxemia and associated inflammatory processes may contribute to progressive alterations of upper airway muscle structure and function, potentially influencing airway stability with increasing age [8].

OSA is diagnosed using either full-night polysomnography (PSG), the diagnostic gold standard, or through home sleep testing with polygraphy (PG) [9]. Disease severity is quantified using the apnea–hypopnea index (AHI), which is defined as the number of apneas and hypopneas per hour of sleep. According to the international consensus criteria, OSA is classified as mild (5 ≤ AHI < 15), moderate (15 ≤ AHI ≤ 30), or severe (AHI > 30) [9,10].

Lateral cephalometry remains a fundamental diagnostic tool in orthodontics, allowing standardized assessment of hard and soft tissue measurements. Although three-dimensional imaging techniques such as CBCT can provide volumetric airway information, their use is constrained by radiation exposure and clinical justification requirements [11]. Consequently, lateral cephalograms continue to represent the most frequently available imaging modality for evaluating airway-related anatomical features in orthodontic patients. Previous studies have investigated associations between OSA and cephalometric airway dimensions, yet results are inconsistent. While some authors reported reduced PAS or airway volumes in individuals with OSA, others found weak or no associations between static airway dimensions and disease presence or severity [12,13]. The interpretation of these findings is limited by substantial methodological heterogeneity, including variable landmark definitions and inconsistent measurement protocols [7]. Importantly, many studies classified control subjects without objective exclusion of OSA using PSG or PG, despite the high prevalence of undiagnosed disease in the general population [12,13,14,15,16]. Beyond PAS, the position of the hyoid bone has been proposed as a relevant anatomical marker in OSA. The hyoid is suspended by a complex muscular and fascial system linking the mandible, tongue base, and cervical spine, and its position is influenced by both craniofacial morphology and neuromuscular tone. An inferiorly positioned hyoid has been reported more frequently in patients with OSA and may reflect increased soft tissue load or altered muscular balance within the upper airway [11,14,17,18,19]. Beyond PAS and hyoid position, soft palate morphology—particularly uvular dimensions—has been discussed as an additional anatomical correlate of OSA. Cephalometric studies have reported increased uvula length (UL) and thickness (UT) in patients with OSA compared with controls, suggesting a higher soft tissue load in the velopharyngeal segment [7]. Mechanistically, repetitive snoring-related vibration and intermittent upper airway obstruction may promote local tissue trauma, inflammation, and edema within the uvular mucosa, potentially contributing to uvular enlargement [20].

However, evidence remains inconsistent, and few studies have simultaneously evaluated PAS, soft palate, and hyoid position with adequate control for confounding factors such as body mass index (BMI) and craniofacial skeletal pattern [21,22,23,24].

Therefore, the aim of this age-matched case–control study was to evaluate whether PAS, uvula length, and thickness, as well as hyoid bone position, assessed on standardized lateral cephalograms differ between individuals with OSA and objectively verified non-OSA controls. As the primary objective, we investigated whether PAS dimensions independently predict OSA severity after adjustment for BMI and sagittal and vertical craniofacial skeletal relationships. As part of secondary exploratory analyses, we examined the predictive value of soft palate and hyoid bone measurements. We hypothesized that static PAS dimensions would not differ significantly between groups, whereas hyoid bone position would be altered in individuals with OSA.

## 2. Material and Methods

### 2.1. Study Design

This case–control study assessed the differences in posterior upper airway and soft palate dimensions, as well as hyoid bone position, between individuals with and without OSA. Ethical approval was obtained from the Ethics Committee of the University of Marburg (approval no. 24-308-BO; approval date: 28 November 2024) in accordance with the Declaration of Helsinki, and the study was registered in the German Clinical Trials Register (DRKS00036018; registered 5 May 2025). Written informed consent was obtained from all patients with and without OSA.

An a priori power analysis was performed using G*Power (version 3.1) [25] based on a two-tailed independent samples *t*-test. The minimum PAS served as the primary endpoint for sample size estimation. On the basis of cephalometric measurements reported by Battagel et al. [26], demonstrating mean values of 8.7 ± 3.0 mm in individuals without OSA and 5.4 ± 3.5 mm in patients with OSA, an expected intergroup difference of 3.4 mm was assumed, corresponding to a standardized effect size of d = 1.01. With a two-sided α level of 0.05 and a target statistical power of 95%, a total sample size of 54 participants was required, comprising 27 individuals per group (allocation ratio 1:1), yielding an actual power of 95.45%.

### 2.2. Study Population

A total of 294 patients diagnosed with OSA who were referred between 2010 and 2025 either by the Interdisciplinary Center of Sleep Medicine at the University of Marburg or by external sleep laboratories to the Department of Orthodontics at the University of Marburg for consultation and treatment were retrospectively screened for eligibility. Screening was performed by a single investigator (JS). The inclusion criteria comprised a confirmed diagnosis of OSA based on overnight polygraphy or polysomnography with a baseline AHI ≥ 5 and the availability of a lateral cephalogram obtained within 6 months of poly(somno)graphic recordings and acquired for orthodontic–orthognathic evaluation.

In parallel, a control cohort was prospectively recruited between 6 May 2025 and 1 November 2025 by JS. Consecutive adult patients initiating orthodontic treatment at the Department of Orthodontics at the University of Marburg, for whom a lateral cephalogram was clinically indicated, were invited to participate. The inclusion criteria for the control group were age (≥18 years), absence of OSA as confirmed by overnight polygraphy (AHI < 5), and availability of a lateral cephalogram acquired for diagnostic orthodontic purposes.

The exclusion criteria for both cohorts included the presence of craniofacial syndromes, a history of major trauma with fractures involving the facial skeleton, and severe injuries to the cervical spine.

After the application of all inclusion and exclusion criteria, the patients with OSA were individually matched to orthodontic controls without OSA based on age.

### 2.3. Cephalometric Measurements

Radiographic acquisition of lateral cephalograms (PLANMECA, ProMax) was performed using standardized exposure parameters ranging from 66 to 68 kV and 5 mA, adjusted according to individual patient size. Head positioning was stabilized using a nasal support aligned to the nasion (N), and orientation was controlled to maintain the Frankfurt horizontal plane. All images were obtained in centric occlusion with the lips in a relaxed position. The cephalograms were originally acquired as part of routine clinical care, either prior to mandibular advancement device (MAD) therapy in subjects with OSA or before the initiation of orthodontic treatment in control subjects.

Prior to analysis, all radiographs were calibrated using a predefined magnification factor. The examiner was blinded to all patient identifiers and group allocation. To ensure measurement reliability, the primary assessor (J.H.) underwent structured training under supervision of an experienced investigator (J.S.). For the assessment of intra-examiner reliability, all cephalograms were re-evaluated by the same examiner after an interval of 2 weeks. Inter-examiner agreement was assessed by independent reanalysis of a random subset of 20 cephalograms by the second investigator (J.S.).

**Figure 1:**
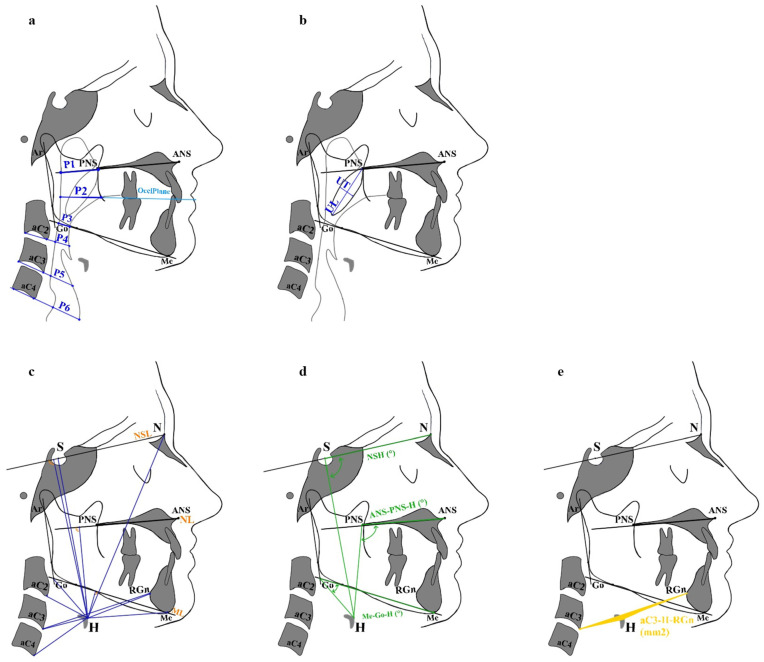
(**a**) Posterior airway space (PAS). Linear assessments were performed at six levels (P1–P6) according to Hourfar et al. [27]. (**b**) Soft palate measurements: uvula length (UL) and uvula thickness (UT). (**c**) Linear measurements from the hyoid bone (H) (HaC2, H-aC3, H-aC4, H-S, H-N, H-Me, H-Go, H-ML, H-NL, and H-NSL). (**d**) Angular measurements from H (N-S-H, ANS-PNS-H, and Me-Go-H). (**e**) Area measurement from H (aC3-H-RGn). Abbreviations are defined in Table 1.

**Figure 2:**
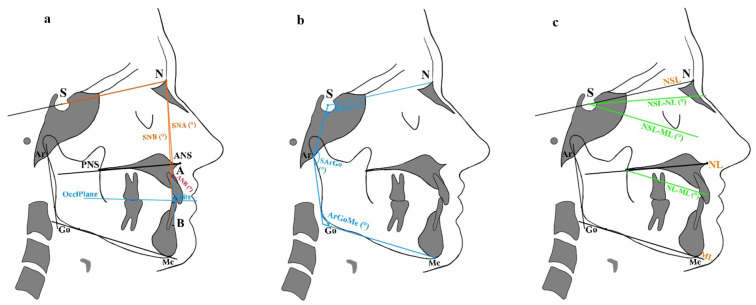
Craniofacial characteristics. (**a**) Linear (Wits appraisal) and angular (ANB) sagittal measurements of intermaxillary relationship. (**b**) Angular vertical characteristics: sum of Björk polygon angles (N-S-Ar, S-Ar-Go, and Ar-Go-Me). (**c**) Angular vertical characteristics: NSL-ML, NL-ML, NSL-ML. Abbreviations are defined in Table 1.

All cephalometric variables included in the analysis are defined in Table 1. A schematic overview of the measurements related to posterior airway space (PAS), soft palate morphology, hyoid bone position, and craniofacial skeletal characteristics is provided in Figure 1 and Figure 2.

### 2.4. Statistical Analysis

Data analysis was performed using IBM SPSS (V. 29.0.2.0). The Shapiro–Wilk test was applied on all continuous variables to test for normality of distribution. Descriptive statistics were calculated for the total study sample for the two study groups. The data are presented with arithmetic means and standard deviations. Group comparisons were performed using independent samples *t*-tests.

To further investigate whether PAS, uvula length and thickness, as well as hyoid variables are associated with OSA severity, multiple linear regression analyses were conducted with the AHI as the continuous dependent variable. All models were adjusted for BMI, ANB, and the sum of Björk polygon angles. Additional sensitivity analyses were performed by including sex as an additional covariate in all regression models. Multicollinearity was assessed using the variance inflation factor (VIF) statistics.

Regression diagnostics were performed for all models. Normality of residuals was assessed using Shapiro–Wilk tests, and influential observations were evaluated using Cook’s distance. Because residuals of the original AHI-based models showed departures from normality, all regression analyses were additionally re-estimated using log-transformed AHI (logAHI) as the dependent variable. The log-transformation improved residual distributions, and all substantive findings remained unchanged. As untransformed AHI values are more directly interpretable in the clinical context, the original models are reported as primary analyses.

The a priori sample size calculation was based on PAS as the primary outcome. Accordingly, PAS-related analyses were defined as the primary endpoint, whereas analyses involving soft palate and hyoid-related variables were considered secondary exploratory analyses.

Intra-rater and inter-rater reliability of the measurements were assessed using intraclass correlation coefficients (ICCs) and their 95% CIs. A *p*-value < 0.05 was considered statistically significant.

## 3. Results

**Figure 3:**
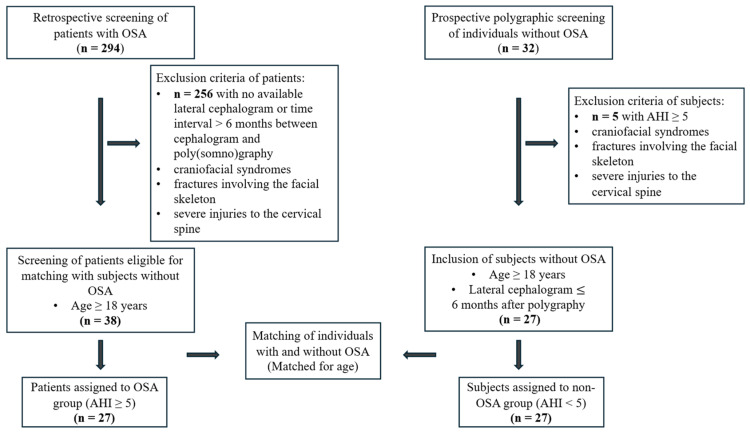
Study flow chart showing screening, exclusion, inclusion, matching, and assignment to groups in the study population. OSA—obstructive sleep apnea, AHI—apnea–hypopnea index, BMI—body mass index.

Of the 32 initially polygraphically screened participants presumed not to have OSA, five exhibited an AHI ≥ 5 and were therefore excluded. After matching participants with and without OSA, a total of 54 individuals were included in the statistical analysis: 27 with OSA (OSA group) and 27 without OSA (non-OSA group) (Figure 3).

The intra- and inter-examiner reliability values showed excellent agreement (ICCintra = 0.96; [CI: 0.91; 0.97]; ICCinter = 0.95; [CI: 0.91; 0.98]) [28].

### 3.1. Sleep Characteristics

Sleep characteristics of the study population are summarized in Table 2.

### 3.2. Demographic Characteristics

Demographic variables for the total study population, as well as between-group comparisons, are summarized in Table 3. Age did not differ significantly between the groups (*p* = 0.228). In contrast, BMI was significantly higher in group 1 (*p* < 0.001), and the distribution of sex also differed significantly between the groups (*p* = 0.002).

**Table 3:** Demographic variables at baseline (t0) for the total study population and for the two groups.

Variables	Total Sample Size (*n* = 54)	OSA Group (*n* = 27)	Non-OSA Group (*n* = 27)	*p*-Value
Age (years) ^a^	35.4 ± 9.9	37.1 ± 8.8	33.8 ± 10.8	0.228
Male sex (%) ^b^	55.6	77.8	33.3	0.002 **
BMI (kg/m^2^) ^a^	25.1 ± 4.4	27.3 ± 3.8	23.1 ± 4.1	<0.001 ***

BMI—body mass index; ^a^ *p*-value was calculated using independent samples *t*-test. ^b^ *p*-value was calculated using fisher’s exact test. ** Statistically significant at *p* < 0.01; *** Statistically significant at *p* < 0.001.

### 3.3. Craniofacial Characteristics, PAS, Soft Palate, and Hyoid Position

Table 4 summarizes sagittal and vertical craniofacial characteristics, PAS, and soft palate and hyoid bone measurements for the total study population and between both groups. No significant differences were found between groups in any sagittal or vertical craniofacial measurements. Similarly, all PAS parameters (P1–P6) showed no significant differences between groups. However, UL and U,T as well as multiple hyoid position measurements, demonstrated significantly higher values in the OSA group. The hyoid differences were observed in H-N, H-S, H-NL, H-NSL, H-Go, H-ML, aC3-H-RGn (mm), aC3-H-RGn (mm^2^), and Me-Go-H (°). Additionally, H-aC2, H-aC3, and H-aC4 exhibited significant differences.

### 3.4. Prediction of OSA Severity Based on PAS Measurements (Table 5)

Multiple regression analyses incorporating PAS variables (P1–P6), BMI, ANB, and the sum of Björk polygon angles demonstrated only modest explanatory power for AHI, with R^2^ values ranging from 0.295 to 0.336 (adjusted R^2^ = 0.151–0.279). Across all six models, BMI consistently emerged as the strongest positive predictor of AHI, showing significant coefficients (B = 0.979–1.167, *p* ≤ 0.031), whereas the PAS measurements exhibited small, non-significant effects (P1: B = −0.361, *p* = 0.305; P2: B = 0.045, *p* = 0.887; P3: B = −0.217, *p* = 0.461; P4: B = −0.553, *p* = 0.097; P5: B = 0.254, *p* = 0.399; P6: B = 0.122, *p* = 0.746). The sum of Björk polygon angles exhibited coefficients ranging from 0.236 to 0.305, with borderline significance observed in model 4 (B = 0.294, *p* = 0.038). ANB demonstrated no significant contribution across any of the models (B = −0.149 to 0.160, all *p* > 0.70). The model fit was statistically supported in five of the six models, with significant F-statistics (F = 4.192–5.934, all *p* ≤ 0.006). However, model 6 failed to reach significance (F (4,40) = 2.026, *p* = 0.131).

**Table 5:** Multiple linear regression analysis (models 1–6) between AHI and PAS (P1–P6), BMI, ANB, and sum of Björk polygon angles as predictors.

Model	Unstandardized Coefficient		Standardized Coefficient	t	*p*
	B	SE B	ß		
**1**					
(Constant)	−100.950	61.452		−1.643	0.107
P1 (mm)	−0.361	0.348	−0.137	−1.036	0.305
BMI (kg/m^2^)	1.087	0.311	0.4554	3.494	0.001 **
ANB (°)	0.160	0.453	0.055	0.354	0.725
Sum of Björk polygon angles (°)	0.236	0.150	0.240	1.579	0.121
R^2^	0.311				
Adjusted R^2^	0.252				
F (df = 4; 54)	5.299				0.001
**2**					
(Constant)	−133.835	60.855		−2.199	0.034 *
P2 (mm)	0.045	0.313	0.019	0.142	0.887
BMI (kg/m^2^)	1.136	0.339	0.474	3.350	0.002 **
ANB (°)	0.051	0.483	0.017	0.106	0.916
Sum of Björk polygon angles (°)	0.293	0.155	0.297	1.886	0.067
R^2^	0.295				
Adjusted R^2^	0.225				
F (df = 4; 54)	4.192				0.006
**3**					
(Constant)	−125.320	54.785		−2.287	0.027 *
P3 (mm)	−0.217	0.291	−0.098	−0.743	0.461
BMI (kg/m^2^)	1.074	0.319	0.449	3.367	0.002 **
ANB (°)	−0.046	0.463	−0.016	−0.099	0.921
Sum of Björk polygon angles (°)	0.283	0.142	0.287	1.998	0.051
R^2^	0.303				
Adjusted R^2^	0.244				
F (df = 4; 54)	5.114				0.002
**4**					
(Constant)	−123.972	53.036		−2.338	0.024 *
P4 (mm)	−0.553	0.327	−0.215	−1.693	0.097
BMI (kg/m^2^)	0.979	0.316	0.409	3.104	0.003 **
ANB (°)	−0.149	0.449	−0.051	−0.332	0.742
Sum of Björk polygon angles (°)	0.294	0.138	0.298	2.131	0.038 *
R^2^	0.336				
Adjusted R^2^	0.279				
F (df = 4; 54)	5.934				<0.001 ***
**5**					
(Constant)	−132.195	55.183		−2.396	0.021 *
P5 (mm)	0.254	0.299	0.106	0.851	0.399
BMI (kg/m^2^)	1.131	0.316	0.472	3.579	0.001 **
ANB (°)	0.082	0.453	0.028	0.181	0.857
Sum of Björk polygon angles (°)	0.284	0.144	0.288	1.969	0.055
R^2^	0.306				
Adjusted R^2^	0.245				
F (df = 4; 54)	4.965				0.002 **
**6**					
(Constant)	−140.696	89.626		−1.570	0.133
P6 (mm)	0.122	0.372	0.067	0.329	0.746
BMI (kg/m^2^)	1.167	0.501	0.487	2.332	0.031 *
ANB (°)	0.053	0.699	0.018	0.075	0.941
Sum of Björk polygon angles (°)	0.305	0.228	0.310	1.341	0.196
R^2^	0.299				
Adjusted R^2^	0.151				
F (df = 4; 54)	2.026				0.131

B—unstandardized coefficient, ß—standardized coefficient; * *p* < 0.05, ** *p* < 0.01, *** *p* < 0.001.

### 3.5. Prediction of OSA Severity Based on Soft Palate Measurements (Table 6)

The regression models incorporating soft palate variables demonstrated modest explanatory power for AHI, with R^2^ values of 0.262 and 0.295 (adjusted R^2^ = 0.199 and 0.235, respectively). In both models, BMI showed a consistent positive association with AHI (model 1: B = 0.951, *p* = 0.003; model 2: B = 0.827, *p* = 0.010).

UL as well as UT were not significantly associated with AHI. ANB did not contribute significantly to either model (model 1: B = −0.190, *p* = 0.670; model 2: B = 0.037, *p* = 0.933). The sum of Björk polygon angle reached statistical significance in the UT model (B = 0.286, *p* = 0.046). Overall model significance was confirmed by the F-statistics for both models (model 1: F(4,54) = 4.163, *p* = 0.006; model 2: F(4,54) = 4.920, *p* = 0.002).

**Table 6:** Multiple linear regression analysis (models 1 and 2) between AHI and uvula length (UL) and thickness (UT), BMI, ANB, and sum of Björk polygon angles as predictors.

Model	Unstandardized Coefficient		Standardized Coefficient	t	*p*
	B	SE B	ß		
**1**					
(Constant)	−117.135	55.578		−2.108	0.040 *
UL (mm)	0.359	0.320	0.145	1.120	0.268
BMI (kg/m^2^)	0.951	0.306	0.415	3.112	0.003 **
ANB (°)	−0.190	0.443	−0.061	−0.429	0.670
Sum of Björk polygon angles (°)	0.233	0.138	0.231	1.682	0.099
R^2^	0.262				
Adjusted R^2^	0.199				
F (df = 4; 54)	4.163				0.006 **
**2**					
(Constant)	−139.429	56.121		−2.484	0.017 *
UT (mm)	1.708	0.907	0.271	1.884	0.066
BMI (kg/m^2^)	0.827	0.310	0.361	2.669	0.010 *
ANB (°)	0.037	0.433	0.012	0.085	0.933
Sum of Björk polygon angles (°)	0.286	0.140	0.264	2.046	0.046 *
R^2^	0.295				
Adjusted R^2^	0.235				
F (df = 4; 54)	4.920				0.002 **

B—unstandardized coefficient, ß—standardized coefficient. * *p* < 0.05, ** *p* < 0.01.

### 3.6. Prediction of OSA Severity Based on Hyoid Measurements (Table 7)

The regression models incorporating hyoid-related variables demonstrated a moderate explanatory capacity for AHI, with R^2^ values ranging from 0.349 to 0.381 (adjusted R^2^ = 0.275–0.328). Across all six models, BMI demonstrated a consistent positive association with AHI (B = 0.833–0.961, *p* = 0.003–0.016). Furthermore, several hyoid measurements were identified as significant predictors. An increased inferior and anterior hyoid position showed a significant association with an higher AHI (H-ML: B = 0.471, *p* = 0.015, H-NSL: B = 0.281, *p* = 0.034, H-aC3: B = 0.628, *p* = 0.020, Me-Go-H: B = 0.363, *p* = 0.020, and aC3-H-RGn: B = 0.012, *p* = 0.045). In contrast, ANB (B = −0.215–0.293, all *p* > 0.500) and the sum of Björk polygon angles (B = 0.224–0.268, all *p* > 0.530) exhibited no significant contribution in any model. The statistical significance of all six models was confirmed by the F-statistics (F = 5.836–7.237, all *p* < 0.001).

However, because sex distribution differed significantly between groups, these findings were further examined in additional sex-adjusted sensitivity analyses (Section 3.7).

**Table 7:** Multiple linear regression analysis (models 1–6) between AHI and hyoid position, BMI, ANB, and sum of Björk polygon angles as predictors.

Model	Unstandardized Coefficient		Standardized Coefficient	t	*p*
	B	SE B	ß		
**1**					
(Constant)	−61.622	52.091		−1.183	0.243
H-ML (mm)	0.471	0.187	0.330	2.516	0.015 *
BMI (kg/m^2^)	0.833	0.294	0.363	2.829	0.007 **
ANB (°)	−0.215	0.417	−0.069	−0.516	0.608
Sum of Björk polygon angles (°)	0.108	0.136	0.107	0.792	0.433
R^2^	0.332				
Adjusted R^2^	0.275				
F (df = 4; 54)	5.836				<0.001 **
**2**					
(Constant)	−145.103	52.220		−2.779	0.008 **
H-NSL (mm)	0.281	0.129	0.295	2.184	0.034 *
BMI (kg/m^2^)	0.824	0.329	0.344	2.507	0.016 *
ANB (°)	0.201	0.430	0.068	0.468	0.642
Sum of Björk polygon angles (°)	0.265	0.136	0.268	1.947	0.058
R^2^	0.360				
Adjusted R^2^	0.306				
F (df = 4; 54)	6.609				<0.001 ***
**3**					
(Constant)	−132.413	52.317		−2.531	0.015 *
H-NL (mm)	0.308	0.156	0.263	1.967	0.055
BMI (kg/m^2^)	0.842	0.334	0.352	2.524	0.015 *
ANB (°)	0.077	0.429	0.026	0.181	0.857
Sum of Björk polygon angles (°)	0.258	0.137	0.262	1.878	0.067
R^2^	0.349				
Adjusted R^2^	0.293				
F (df = 4; 54)	6.290				<0.001 ***
**4**					
(Constant)	−138.259	51.425		−2.689	0.010 *
H-aC3 (mm)	0.628	0.261	0.320	2.407	0.020 *
BMI (kg/m^2^)	0.845	0.317	0.353	2.666	0.011 *
ANB (°)	0.293	0.432	0.100	0.679	0.501
Sum of Björk polygon angles (°)	0.266	0.134	0.270	1.982	0.053
R^2^	0.372				
Adjusted R^2^	0.319				
F (df = 4; 54)	6.972				<0.001 ***
**5**					
(Constant)	−111.400	52.049		−2.689	0.010 *
Me-Go-H (°)	0.363	0.151	0.300	2.404	0.020 *
BMI (kg/m^2^)	0.933	0.305	0.390	3.054	0.004 **
ANB (°)	−0.054	0.423	−0.018	−0.128	0.899
Sum of Björk polygon angles (°)	0.224	0.137	0.227	1.639	0.108
R^2^	0.372				
Adjusted R^2^	0.319				
F (df = 4; 54)	6.967				<0.001 ***
**6**					
(Constant)	−123.469	52.279		−2.363	0.022 *
aC3-H-RGn (mm^2^)	0.012	0.006	0.255	2.059	0.045 *
BMI (kg/m^2^)	0.961	0.310	0.401	3.102	0.003 **
ANB (°)	0.125	0.428	0.043	0.293	0.771
Sum of Björk polygon angles (°)	0.268	0.136	0.272	1.967	0.055
R^2^	0.353				
Adjusted R^2^	0.298				
F (df = 4; 54)	6.421				<0.001 ***

B—unstandardized coefficient, ß—standardized coefficient; * *p* < 0.05, ** *p* < 0.01, *** *p* < 0.001.

### 3.7. Sensitivity Analyses Adjusted for Sex

Because sex distribution differed significantly between groups, additional sensitivity analyses were performed by including sex as a covariate in all hyoid-based regression models. After adjustment for sex, the previously significant associations between hyoid-related variables and AHI were attenuated and no longer statistically significant for H-ML (B = 0.292, *p* = 0.160), H-NSL (B = 0.080, *p* = 0.629), H-NL (B = 0.026, *p* = 0.896), H-aC3 (B = −0.145, *p* = 0.663), Me-Go-H (B = 0.238, *p* = 0.097), and aC3-H-RGn (B = 0.098, *p* = 0.680) Sex emerged as a significant covariate in models 4–6 (H-aC3: *p* = 0.015; Me-Go-H: *p* = 0.018; aC3-H-RGn: *p* = 0.036). BMI remained a significant predictor in all models (all *p* ≤ 0.049). The sum of Björk polygon angles became significant in models 3, 4, and 6 (*p* = 0.045, *p* = 0.034, *p* = 0.044, respectively) after sex adjustment. Detailed results of the sex-adjusted hyoid models are provided in the Supplementary Materials (Table S1).

## 4. Discussion

In this age-matched case–control study, we investigated whether PAS and hyoid position differ between individuals with and without OSA and whether these parameters independently predict disease presence.

Among the available cephalometric studies on OSA, to our knowledge, limited evidence exists from case–control designs that objectively verified the absence of OSA in the control group using polygraphy. This methodological approach enabled the identification and exclusion of five individuals who were clinically asymptomatic but were incidentally diagnosed with previously unrecognized OSA. This substantially reduced the risk of false-negative classification. In addition, we adjusted for key confounding factors known to influence both airway anatomy and disease severity, most notably BMI, as well as sagittal and vertical craniofacial skeletal relationships, which could also affect both upper airway caliber and hyoid position. This comprehensive adjustment strengthens the validity of the observed associations.

Consistent with these methodological considerations, PAS measurements derived from lateral cephalograms did not differ between subjects with OSA and objectively verified controls, and none of the PAS parameters independently predicted AHI after multivariable adjustment. In all PAS-based models, BMI emerged as the strongest predictor of AHI. These findings suggest that static sagittal airway dimension assessed on lateral cephalograms does not provide meaningful information for OSA risk stratification when BMI and craniofacial morphology are appropriately accounted for.

Soft palate measurements differed significantly between groups: both UL and UT were higher in subjects with OSA. These findings align with prior cephalometric reports describing soft palate/uvula enlargement in OSA, which has been attributed to chronic vibration-related tissue trauma with inflammatory remodeling and edema [7,20]. However, despite the intergroup differences, neither UL nor UT independently predicted AHI after adjustment for BMI and craniofacial skeletal pattern. This suggests that uvular enlargement may reflect OSA-associated soft tissue changes and/or confounding by adiposity rather than providing incremental value for explaining disease severity.

In contrast, hyoid-related parameters differed significantly between groups and several remained independently associated with AHI after adjustment for BMI and skeletal pattern. Among these, the distance from the hyoid to the mandibular plane (H-ML) demonstrated the most consistent association. Together, these findings suggest that static cephalometric airway space is not an informative marker for OSA risk, whereas hyoid position may provide incremental anatomical information when imaging is already clinically indicated for orthodontic or surgical indications. The hyoid bone is suspended by a complex muscular and fascial network. An inferior position likely reflects increased soft tissue load and altered neuromuscular balance within the upper airway, potentially contributing to retroglossal collapsibility during sleep. The observed associations between inferior (and partially anterior) hyoid position and higher AHI are consistent with prior cephalometric studies linking mandibular plane-to-hyoid distance with OSA risk and severity [7].

A notable strength of our analytical approach is the adjustment for craniofacial skeletal morphology. By including sagittal (ANB) and vertical (sum of Björk polygon angles) parameters, we accounted for dentofacial pattern as a potential source of variation in hyoid posture. The persistence of associations for several hyoid metrics supports the interpretation that hyoid position contributes information beyond skeletal relationship in this cohort. Furthermore, the association between AHI and the aC3-H-RGn area suggests that geometric configuration of the hyoid relative to the cervical spine and mandibular symphysis may capture clinically relevant aspects of tongue base–neck anatomy.

However, these associations were attenuated in the additional sensitivity analyses after adjustment for sex. In the unadjusted models, H-ML (*p* = 0.015), H-NSL (*p* = 0.034), H-aC3 (*p* = 0.020), Me-Go-H (*p* = 0.020), and aC3-H-RGn (*p* = 0.045) all reached significance, whereas none of these variables remained significant after sex adjustment. At the same time, sex itself emerged as a significant or near-significant covariate in most models, with negative coefficients indicating lower AHI values in female participants. This pattern suggests that the observed hyoid–AHI associations in the unadjusted models may have been partly confounded by sex-related differences in craniofacial anatomy and OSA susceptibility and should be interpreted cautiously and regarded as exploratory rather than definitive independent predictors of OSA severity.

These observations are consistent with findings by a recent meta-analysis that confirmed that the hyoid bone–mandibular plane distance is significantly influenced by sex in individuals with OSA, with males exhibiting substantially greater values than females [29]. Furthermore, pharyngeal length is generally known to be longer in males compared to females, which has been proposed as a contributing factor to sex differences in upper airway collapsibility and OSA prevalence [30].

BMI was a dominant contributor across all models and remained strongly associated with AHI in both PAS- and hyoid-based analyses. These findings reinforce the central role of adiposity in OSA pathogenesis, including increased soft tissue load in the tongue and parapharyngeal region, reduced upper-airway caliber during sleep, and heightened collapsibility. From a methodological perspective, these findings highlight BMI as a significant confounding factor in craniofacial imaging research. Studies reporting associations between smaller static airway dimensions (whether on cephalograms or CBCT) and OSA may partially reflect BMI imbalance if adiposity is not adequately controlled. In the present analyses, adjustment for BMI effectively eliminated any apparent association between PAS and OSA, suggesting that reports of reduced static airway dimensions may partly reflect unaccounted weight-related effects.

### 4.1. Strengths and Limitations

This study addresses several limitations of previous research. First, OSA was defined using established AHI criteria, and the control group was objectively verified as non-OSA by polygraphy, thereby substantially reducing misclassification bias. Second, participants were age-matched, and cephalograms were obtained in temporal proximity to sleep studies, strengthening the comparability between anatomical and physiological measurements. Third, multivariable regression models accounted for BMI, as well as sagittal and vertical craniofacial skeletal relationships, and additional sensitivity analyses were performed to evaluate the influence of sex.

Measurement reliability was enhanced through standardized imaging protocols, blinded assessment, repeated measurements, and evaluation of intra- and inter-rater reliability. PAS was assessed using a standardized multi-level approach, and highly mobile soft tissue landmarks were deliberately avoided [27,31,32,33].

Several limitations warrant consideration. Lateral cephalograms provide two-dimensional representations of a three-dimensional upper airway and do not allow assessment of transverse dimensions or volumetric airway characteristics. Although a previous study has demonstrated good agreement between linear PAS measurements obtained from lateral cephalograms and those derived from computed tomography or cone beam computed tomography, three-dimensional aspects of airway morphology could not be evaluated in the present study [34].

In addition, lateral cephalograms are acquired during wakefulness and represent static images obtained without the standardization of respiratory phase. Upper airway dimensions are known to vary across the respiratory cycle, and this physiological variability may contribute to measurement variability and limit comparability across individuals [35,36].

Moreover, although the mean ANB values in both groups fall within the skeletal class I range, the considerable standard deviations and the wide spread of Wits appraisal values indicate that the sample was not exclusively composed of class I subjects but included individuals with skeletal class II and III. Nevertheless, the predominance of class I skeletal patterns in the study population may limit the generalizability of the present findings.

The mixed retrospective–prospective case–control design should also be considered a limitation. OSA cases were retrospectively identified from referred patients between 2010 and 2025, whereas controls were prospectively recruited from orthodontic patients in 2025. Because these groups arose from different source populations and referral pathways, residual differences beyond OSA status cannot be excluded. Moreover, controls were not population-based non-OSA adults but orthodontic patients with clinically indicated cephalograms.

Furthermore, the significant sex imbalance between groups (77.8% male in the OSA group vs. 33.3% in the non-OSA group) represents a relevant limitation. Although sensitivity analyses adjusting for sex were performed, the limited sample size precluded reliable sex-stratified subgroup analyses or interaction modeling. Given well-established sex-related differences in OSA susceptibility, future studies with larger, sex-balanced cohorts are warranted to disentangle sex-specific effects from hyoid-related anatomical contributions.

Moreover, the present study employed multiple regression models in a relatively small sample (*n* = 54), which inherently increases the risk of overfitting and may limit the stability of the reported parameter estimates. Although the PAS-related analyses were pre-specified as the primary analyses, and the number of predictors per model was restricted, the overall analytical framework also included several secondary and exploratory regression models with partially overlapping predictor sets. No formal correction for multiple testing was applied, and the risk of type I error inflation cannot therefore be excluded. Accordingly, findings from secondary analyses, particularly those related to hyoid parameters, should be regarded as hypothesis-generating rather than confirmatory. In addition, the limited sample size may have contributed to model instability, such that small perturbations in the data could lead to meaningful changes in parameter estimates, further limiting the generalizability of the reported associations. Future studies with larger samples are needed to confirm these findings and to assess their robustness across different skeletal patterns.

### 4.2. Clinical Implications

Lateral cephalograms are routinely obtained in orthodontic and surgical dentofacial orthopedic practice for assessment of skeletal relationships and treatment planning. Airway dimensions derived from these images are frequently interpreted as indicators of OSA risk. The present findings indicate that static PAS measurements on lateral cephalograms provide limited information regarding OSA presence or severity and should not be used for OSA screening or risk stratification. Because lateral cephalograms represent static, wake-state acquisitions, they do not capture sleep-related changes in neuromuscular tone or pharyngeal collapsibility. Accordingly, there is no justification for obtaining cephalometric imaging solely to estimate OSA risk. Radiographic examinations should remain restricted to established dentofacial indications and should be interpreted cautiously in the context of suspected sleep-disordered breathing.

In contrast, hyoid bone position—particularly its vertical relationship to the mandibular plane—may provide complementary anatomical information when a lateral cephalogram is already clinically indicated. However, hyoid-related measurements should be considered in conjunction with established clinical risk factors and should not be interpreted as standalone diagnostic markers.

When OSA is suspected, validated clinical screening tools and referral to sleep medicine for objective diagnostic testing remain essential. Further studies are required to determine whether cephalometric hyoid parameters have prognostic or therapeutic relevance in orthodontic or surgical treatment planning for patients with OSA.

## 5. Conclusions

• PAS measurements on lateral cephalograms were not associated with OSA and did not predict AHI after multivariable adjustment, providing no rationale for PAS-based OSA risk assessment.
• Although UL and UT differed significantly between groups, they were not independent predictors of AHI, and therefore do not support uvula-based OSA screening.
• BMI emerged as the strongest predictor of OSA severity, underscoring its central role in established screening tools.
• Several hyoid-related variables were associated with AHI in the primary models; however, these associations were attenuated after additional adjustment for sex and should therefore be interpreted cautiously as exploratory findings, pending external validation.
• In dental settings, OSA screening should rely primarily on validated questionnaires and established clinical risk factors rather than static cephalometric airway measurements.

## Figures and Tables

**Table 1:** Definition of cephalometric measurements.

**Measurement**	**I. PAS**
**Linear (mm)**	
P1	Distance between the posterior pharyngeal wall and anterior pharyngeal wall or tongue base at the palatal level.
P2	Distance between the posterior pharyngeal wall and anterior pharyngeal wall or tongue base at the level of the occlusal plane.
P3	Distance between the posterior pharyngeal wall and anterior pharyngeal wall or tongue base at the level of the second cervical vertebra.
P4	Distance between the posterior pharyngeal wall and anterior pharyngeal wall at the mandibular plane level.
P5	Distance between the posterior pharyngeal wall and anterior pharyngeal wall at the level of the third cervical vertebra.
P6	Distance between the posterior pharyngeal wall and anterior pharyngeal wall at the level of the fourth cervical vertebra.
**Measurement**	**II. Soft Palate**
**Linear (mm)**	
UL	Length of the soft palate (including the uvula).
UT	Maximum thickness of the soft palate (including the uvula).
**Measurement**	**III. Hyoid Bone**
**Linear (mm)**	
H-aC2	Distance from the most anterior–superior point of the hyoid bone (=H) to the anterior margin of the second cervical vertebral body (=aC2).
H-aC3	Distance from the most anterior–superior point of the hyoid bone (=H) to the anterior margin of the third cervical vertebral body (=aC3).
H-aC4	Distance from the most anterior–superior point of the hyoid bone (=H) to the anterior margin of the fourth cervical vertebral body (=aC4).
H-N	Distance from the most anterior–superior point of the hyoid bone (=H) to the nasofrontal suture intersection in the mid-sagittal plane (=N).
H-S	Distance from the most anterior–superior point of the hyoid bone (=H) to the midpoint of the sella turcica (=S).
H-NL	Perpendicular distance from the most anterior–superior point of the hyoid bone (=H) to the palatal plane (=NL, defined by the line connecting the anterior nasal spine (=ANS) and the posterior nasal spine (=PNS)).
H-NSL	Perpendicular distance from the most anterior–superior point of the hyoid bone (=H) to the anterior cranial base (=NSL, defined by the line through N and S).
H-ML	Perpendicular distance from the most anterior–superior point of the hyoid bone (=H) to the mandibular plane (=ML, defined by the line through Go and Me).
H-Go	Distance from the most anterior–superior point of the hyoid bone (=H) to gonion (Go), the most posterior–inferior point of the mandibular angle constructed at the intersection of the posterior and inferior borders of the mandibular ramus (=Go).
H-Me	Distance from the most anterior–superior point of the hyoid bone (=H) to the most caudal point of the symphysis (=Me).
H-RGn	Distance from the most anterior–superior point of the hyoid bone (=H) to the most posterior point on the contour of the mandibular symphysis (=RGn).
H-RGn-aC3	Perpendicular distance from the most anterior–superior point of the hyoid bone (=H) to the RGn-aC3 plane, defined as a line connecting retrognathion (RGn) and the anterior margin of the third cervical vertebra (aC3).
**Area (mm^2^)**	
aC3-H-RGn	Triangular area enclosed by the most anterior–superior point of the hyoid bone (H), the anterior margin of the third cervical vertebral body (aC3), and retrognathion (RGn).
**Angular (°)**	
Me-Go-H	Angle formed at gonion (Go) between menton (Me), the most posterior–inferior point of the mandibular angle at the junction of the posterior and inferior ramus borders, and the most anterior–superior point of the hyoid bone (H).
ANS-PNS-H	Angle formed at the posterior nasal spine (PNS) between the anterior nasal spine (=ANS) and the most anterior–superior point of the hyoid bone (H).
N-S-H	Angle formed at sella (S), the midpoint of the pituitary fossa, between nasion (N) at the nasofrontal suture intersection in the mid-sagittal plane and the most anterior–superior point of the hyoid bone (=H).
**Measurement**	**IV. Craniofacial Characteristics**
**Linear (mm)**	
Wits appraisal	Distance between the perpendicular projections of A-point (=the deepest concavity on the anterior maxillary surface) and B-point (=the deepest concavity on the anterior mandibular contour) onto the occlusal plane (OcclPl).
**Angular (°)**	
ANB	Sagittal jaw relationship expressed as the angular difference between SNA (=sella–nasion to A-point) and SNB (sella–nasion to B-point).
Sum of Björk polygon angles	The combined total of the saddle angle (N-S-Ar), the articular angle (S-Ar-Go), and the gonial angle (Ar-Go-Me)
NSL/ML	Angular relationship between the mandibular plane (ML) and the anterior cranial base line (nasion–sella line, NSL).
NSL/NL	Angular relationship between the palatal plane (NL) and the anterior cranial base (NSL).
NL/ML	Vertical relationship between the nasal line (NL) and the mandibular line (ML).

**Table 2:** Apnea–hypopnea index (AHI) of total sample size and between subjects with and without OSA.

Variables	Total Sample Size (*n* = 54)	OSA Group (*n* = 27)	Non-OSA Group (*n* = 27)	*p*-Value ^a^
AHI	9.9 ± 10.6	17.7 ± 10.0	2.0 ± 1.5	<0.001 ***

OSA—obstructive sleep apnea, AHI—apnea–hypopnea index; ^a^ *p*-value was calculated using independent samples *t*-test. *** Statistically significant at *p* < 0.001.

**Table 4:** Sagittal and vertical craniofacial characteristics, posterior airway space (PAS), soft palate and hyoid bone measurements for the total study population and between both groups.

Variables	Total Sample Size (*n* = 54)	OSA Group (*n* = 27)	Non-OSA Group (*n* = 27)	*p*-Value ^a^
**Sagittal plane**				
ANB (°)	2.3 ± 3.6	2.2 ± 3.2	2.4 ± 4.0	0.838
SNA (°)	81.4 ± 4.3	80.7 ± 4.4	82.0 ± 4.2	0.274
SNB (°)	79.0 ± 6.2	78.5 ± 6.0	79.6 ± 6.4	0.492
Wits appraisal (mm)	−1.1 ± 4.5	−1.5 ± 4.1	−0.7 ± 4.9	0.512
**Vertical plane**				
Sum of Björk polygon angles (°)	389.8 ± 5.0	392.2 ± 10.3	387.4 ± 10.9	0.099
NSL-ML (°)	30.6 ± 11.1	32.7 ± 11.0	28.5 ± 10.9	0.172
NSL-NL (°)	8.7 ± 4.6	9.3 ± 4.9	8.1 ± 4.3	0.361
NL-ML (°)	22.7 ± 9.9	24.2 ± 10.3	21.2 ± 9.5	0.258
Jarabak ratio (mm)	69.1 ± 8.7	67.7 ± 7.3	70.5 ± 9.9	0.238
**Posterior airway space**				
P1 (mm)	24.9 ± 4.0	23.9 ± 3.9	25.9 ± 3.9	0.067
P2 (mm)	20.3 ± 4.6	20.2 ± 3.8	20.4 ± 5.3	0.841
P3 (mm)	9.5 ± 4.8	8.3 ± 3.7	9.8 ± 5.7	0.237
P4 (mm)	9.1 ± 4.1	8.1 ± 3.5	10.0 ± 4.5	0.093
P5 (mm)	11.0 ± 4.4	11.4 ± 4.5	10.7 ± 4.4	0.558
P6 (mm)	17.6 ± 5.8	15.4 ± 4.8	19.3 ± 6.0	0.094
**Soft palate**				
UL (mm)	34.3 ± 0.6	35.7 ± 4.0	32.9 ± 3.7	0.008 **
UT (mm)	9.8 ± 0.2	10.5 ± 1.5	9.0 ± 1.4	<0.001 ***
**Hyoid position**				
H-aC2 (mm)	40.0 ± 6.7	42.7 ± 5.2	37.4 ± 7.0	0.002 **
H-aC3 (mm)	35.6 ± 5.4	38.1 ± 4.5	33.1 ± 5.2	<0.001 ***
H-aC4 (mm)	39.8 ± 4.9	41.3 ± 4.9	38.5 ± 4.7	0.048 *
H-N (mm)	130.0 ± 12.1	137.0 ± 10.1	123.10 ± 9.6	<0.001 ***
H-S (mm)	109.7 ± 11.4	116.0 ± 7.9	103.43 ± 10.9	<0.001 ***
H-NL (mm)	66.0 ± 9.1	71.0 ± 6.5	61.1 ± 8.7	<0.001 ***
H-NSL (mm)	109.1 ± 11.1	115.1 ± 7.7	103.1 ± 10.8	<0.001 ***
H-ML (mm)	18.3 ± 7.4	22.5 ± 6.7	14.2 ± 5.5	<0.001 ***
H-Go (mm)	37.2 ± 7.5	40.4 ± 7.6	33.9 ± 6.0	0.001 **
H-Me (mm)	44.3 ± 6.4	44.7 ± 5.4	44.0 ± 7.4	0.688
H-RGn (mm)	39.4 ± 6.0	40.6 ± 4.9	38.3 ± 6.8	0.151
aC3-H-RGn (mm)	10.2 ± 6.3	13.2 ± 5.2	7.2 ± 5.8	<0.001 ***
aC3-H-RGn (mm^2^)	368.7 ± 232.4	484.0 ± 149.5	253.4 ± 211.3	<0.001 ***
Me-Go-H (°)	29.1 ± 8.8	33.7 ± 8.0	24.5 ± 6.9	<0.001 ***
ANS-PNS-H (°)	95.7 ± 6.2	95.3 ± 7.0	96.0 ± 5.4	0.692
NSH (°)	90.8 ± 5.6	92.1 ± 6.3	89.5 ± 4.6	0.089

^a^ *p*-value was calculated using independent samples *t*-test. * *p* < 0.05, ** *p* < 0.01, *** *p* < 0.001.

## Data Availability

The original contributions presented in this study are included in the article. Further inquiries can be directed to the corresponding author.

## Supplementary Materials

The following supporting information can be downloaded at: https://www.mdpi.com/article/10.3390/diagnostics16081130/s1, Table S1. Sensitivity analysis: Multiple linear regression models (1–6) between AHI and hyoid position, adjusted for BMI, sex, ANB, and sum of Björk polygon angles.

## References

[1] Benjafield A.V., Pepin J.-L., Cistulli P.A., Wimms A., Lavergne F., Kuniyoshi F.H.S., Munson S.H., Schuler B., Badikol S.R., Wolfe K.C. (2025). Positive airway pressure therapy and all-cause and cardiovascular mortality in people with obstructive sleep apnoea: A systematic review and meta-analysis of randomised controlled trials and confounder-adjusted, non-randomised controlled studies. Lancet Respir. Med..

[2] Benjafield A.V., Ayas N.T., Eastwood P.R., Heinzer R., Ip M.S.M., Morrell M.J., Nunez C.M., Patel S.R., Penzel T., Pépin J.-L. (2019). Estimation of the global prevalence and burden of obstructive sleep apnoea: A literature-based analysis. Lancet Respir. Med..

[3] Vaienti B., Di Blasio M., Arcidiacono L., Santagostini A., Di Blasio A., Segù M. (2024). A narrative review on obstructive sleep apnoea syndrome in paediatric population. Front. Neurol..

[4] Gottlieb D.J., Punjabi N.M. (2020). Diagnosis and Management of Obstructive Sleep Apnea: A Review. JAMA.

[5] Turnbull C.D., Stradling J.R. (2023). Endotyping, phenotyping and personalised therapy in obstructive sleep apnoea: Are we there yet?. Thorax.

[6] Hartfield P.J., Janczy J., Sharma A., Newsome H.A., Sparapani R.A., Rhee J.S., Woodson B.T., Garcia G.J. (2023). Anatomical determinants of upper airway collapsibility in obstructive sleep apnea: A systematic review and meta-analysis. Sleep Med. Rev..

[7] Finke H., Drews A., Engel C., Koos B. (2024). Craniofacial risk factors for obstructive sleep apnea—Systematic review and meta-analysis. J. Sleep Res..

[8] Degerli M.A., Koehler U., Kesper K., Hildebrandt O., Conradt R., Koehler N., Stenger M., Hildebrandt W., Sambale J. (2024). Der obere Atemweg bei OSA-Patienten ist auch im Wachzustand pathologisch. Pneumologie.

[9] Kapur V.K., Auckley D.H., Chowdhuri S., Kuhlmann D.C., Mehra R., Ramar K., Harrod C.G. (2017). Clinical Practice Guideline for Diagnostic Testing for Adult Obstructive Sleep Apnea: An American Academy of Sleep Medicine Clinical Practice Guideline. J. Clin. Sleep Med..

[10] American Academy of Sleep Medicine (2014). International Classification of Sleep Disorders.

[11] American Academy of Oral and Maxillofacial Radiology (2013). Clinical recommendations regarding use of cone beam computed tomography in orthodontics. Position statement by the American Academy of Oral and Maxillofacial Radiology. Oral Surg. Oral Med. Oral Pathol. Oral Radiol..

[12] Taha Y.M., Abu El Sadat S.M., Gaber R.M., Farid M.M. (2025). Ability of upper airway metrics to predict obstructive sleep apnea severity: A systematic review. Dentomaxillofac. Radiol..

[13] Gurgel M.L., Junior C.C., Cevidanes L.H.S., Silva P.G.d.B., Carvalho F.S.R., Kurita L.M., Cunha T.C.A., Fabbro C.D., Costa F.W.G. (2023). Methodological parameters for upper airway assessment by cone-beam computed tomography in adults with obstructive sleep apnea: A systematic review of the literature and meta-analysis. Sleep Breath..

[14] Wei Z., Zhao T., Li Y., Ngan P., Wang Z., Hua F., He H. (2025). The dentofacial and upper airway morphology of adults with obstructive sleep apnea: A systematic review and meta-analysis. Sleep Med. Rev..

[15] Kazmouz S., Calzadilla N., Choudhary A., McGinn L.S., Seaman A., Purnell C.A. (2025). Radiographic findings predictive of obstructive sleep apnea in adults: A systematic review and meta-analysis. J. Cranio-Maxillofac. Surg..

[16] Bayat M., Shariati M., Rakhshan V., Abbasi M., Fateh A., Sobouti F., Davoudmanesh Z. (2017). Cephalometric risk factors of obstructive sleep apnea. CRANIO^®^.

[17] Fagundes N.C.F., Gianoni-Capenakas S., Heo G., Flores-Mir C. (2022). Craniofacial features in children with obstructive sleep apnea: A systematic review and meta-analysis. J. Clin. Sleep Med..

[18] Au C.T., Chan K.C.C., Liu K.H., Chu W.C.W., Wing Y.K., Li A.M. (2018). Potential Anatomic Markers of Obstructive Sleep Apnea in Prepubertal Children. J. Clin. Sleep Med..

[19] Parekh M.H., Thuler E., Triantafillou V., Seay E., Sehgal C., Schultz S., Keenan B.T., Schwartz A.R., Dedhia R.C. (2024). Physiologic and anatomic determinants of hyoid motion during drug-induced sleep endoscopy. Sleep Breath..

[20] Koehler U., Hildebrandt O., Degerli M.A., Viniol C., Hildebrandt W., Conradt R., Birk R., Stuck B., Sambale J., Korbmacher-Steiner H. (2025). Vom Vibrationstrauma zur pharyngealen Muskelinstabilität—Ein sich selbst unterhaltender pathophysiologischer Prozess (Circulus vitiosus) bei obstruktiver Schlafapnoe (OSA). Pneumologie.

[21] Alessandri-Bonetti A., Bortolotti F., Moreno-Hay I., Michelotti A., Cordaro M., Alessandri-Bonetti G., Okeson J.P. (2019). Effects of mandibular advancement device for obstructive sleep apnea on temporomandibular disorders: A systematic review and meta-analysis. Sleep Med. Rev..

[22] Camañes-Gonzalvo S., Bellot-Arcís C., Marco-Pitarch R., Montiel-Company J.M., García-Selva M., Agustín-Panadero R., Paredes-Gallardo V., Puertas-Cuesta F.J. (2022). Comparison of the phenotypic characteristics between responders and non-responders to obstructive sleep apnea treatment using mandibular advancement devices in adult patients: Systematic review and meta-analysis. Sleep Med. Rev..

[23] Paddenberg-Schubert E., Holmer B., Krohn S., Hösl H., Proff P., Kirschneck C., Arzt M. (2025). Predictors of disease alleviation with mandibular advancement devices in obstructive sleep apnea: A retrospective cohort study. Head Face Med..

[24] Indriksone I., Jakobsone G. (2014). The upper airway dimensions in different sagittal craniofacial patterns: A systematic review. Stomatologija.

[25] Faul F., Erdfelder E., Lang A.-G., Buchner A. (2007). G*Power 3: A flexible statistical power analysis program for the social, behavioral, and biomedical sciences. Behav. Res. Methods.

[26] Battagel J. (2000). A cephalometric comparison of subjects with snoring and obstructive sleep apnoea. Eur. J. Orthod..

[27] Hourfar J., Kinzinger G.S.M., Meißner L.K., Lisson J.A. (2017). Effects of two different removable functional appliances on depth of the posterior airway space: A retrospective cephalometric study. J. Orofac. Orthop..

[28] Koo T.K., Li M.Y. (2016). A Guideline of Selecting and Reporting Intraclass Correlation Coefficients for Reliability Research. J. Chiropr. Med..

[29] Graizel-Armoni D., Greenbaum T., Emodi-Perlman A., Koos B. (2025). Sex difference in the hyoid bone position in adults with obstructive sleep apnea: Systematic review and meta-analysis. Dent. Med. Probl..

[30] Jo J.H., Park J.W., Jang J.H., Chung J.W. (2022). Hyoid bone position as an indicator of severe obstructive sleep apnea. BMC Pulm. Med..

[31] Kinzinger G., Czapka K., Ludwig B., Glasl B., Gross U., Lisson J. (2011). Effects of fixed appliances in correcting Angle Class II on the depth of the posterior airway space: FMA vs. Herbst appliance—A retrospective cephalometric study. J. Orofac. Orthop..

[32] Tepedino M., Illuzzi G., Laurenziello M., Perillo L., Taurino A.M., Cassano M., Guida L., Burlon G., Ciavarella D. (2022). Craniofacial morphology in patients with obstructive sleep apnea: Cephalometric evaluation. Braz. J. Otorhinolaryngol..

[33] Bock N.C., Sonntag G., Klaus K., Ruf S. (2025). Posterior airway changes during and after Herbst appliance treatment. Clin. Oral Investig..

[34] Ashique Abdulhameed S., Riyaz A.K., Almutairy M., Khan N.S., Jayakumar S., Gaonkar P. (2024). Assessing the Accuracy of Lateral Cephalogram in Quantifying Three-Dimensional Pharyngeal Airway Morphology Compared to Cone-Beam Computed Tomography. Cureus.

[35] Schwab R.J., Gupta K.B., Gefter W.B., Metzger L.J., Hoffman E.A., Pack A.I. (1995). Upper airway and soft tissue anatomy in normal subjects and patients with sleep-disordered breathing. Significance of the lateral pharyngeal walls. Am. J. Respir. Crit. Care Med..

[36] Chen N., Li K.K., Li S., Wong C., Chuang M., Hwang C., Wu Y. (2002). Airway Assessment by Volumetric Computed Tomography in Snorers and Subjects With Obstructive Sleep Apnea in a Far-East Asian Population (Chinese). Laryngoscope.

