# Supplementary material for: Craniofacial Analysis of Lateral Cephalograms in Obstructive Sleep Apnea—An Exploratory Case–Control Study
Source: Diagnostics (Basel). 2026 Apr 9;16(8):1130. doi: 10.3390/diagnostics16081130 (PMC13114340; doi:10.3390/diagnostics16081130)
Supplement: Supplementary file 1 [file diagnostics-16-01130-s001.zip › diagnostics-4209620-supplementary.pdf]

Table S1. Sensitivity analysis: Multiple linear regression models (1–6) between AHI and hyoid position, adjusted for BMI, sex, ANB, and sum of Björk polygon angles.

	Unstandardized coefficient		Standardized coefficient		
**Model**	**B**	**SE B**	**ß**	**t**	**p**
**1**					
(Constant)	-82.718	51.818		-1.596	0.117
H-ML (mm)	0.292	0.204	0.205	1.429	0.160
BMI (kg/m²)	0.645	0.302	0.281	2.132	0.038*
Sex	-5.884	3.053	-0.290	-1.927	0.060
ANB (°)	-0.063	0.413	-0.020	-0.152	0.880
Sum of Björk polygon angles (°)	0.203	0.141	0.202	1.439	0.157
R^2	0.382				
adjusted R^2	0.315				
F (df = 4; 54)	5.681				<0.001***
**2**					
(Constant)	-111.530	50.522		-2.208	0.032*
H-NSL (mm)	0.080	0.165	0.090	0.487	0.629
BMI (kg/m²)	0.639	0.316	0.278	2.021	0.049*
Sex	-6.612	3.787	-0.326	-1.746	0.088
ANB (°)	0.059	0.416	0.019	0.142	0.888
Sum of Björk polygon angles (°)	0.271	0.135	0.269	2.007	0.051
R^2	0.358				
adjusted R^2	0.288				
F (df = 4; 54)	5.122				<0.001***
**3**					
(Constant)	-107.592	49.944		-2.154	0.036*
H-NL (mm)	0.026	0.199	0.024	0.132	0.896
BMI (kg/m²)	0.665	0.316	0.290	2.103	0.041*
Sex	-7.527	3.797	-0.371	-1.982	0.053
ANB (°)	0.037	0.418	0.012	0.088	0.930
Sum of Björk polygon angles (°)	0.281	0.136	0.279	2.057	0.045*
R^2	0.355				
adjusted R^2	0.284				
F (df = 4; 54)	5.054				0.001**
**4**					
(Constant)	-102.565	51.027		-2.010	0.050
H-aC3 (mm)	-0.145	0.330	-0.074	-0.439	0.663
BMI (kg/m²)	0.696	0.312	0.303	2.232	0.031*

Sex	-8.805	3.501	-0.434	-2.515	0.015*
ANB (°)	0.018	0.418	0.006	0.043	0.966
Sum of Björk polygon angles (°)	0.288	0.132	0.286	2.185	0.034*
R^2	0.357				
adjusted R^2	0.287				
F (df = 4; 54)	5.108				<0.001***
**5**					
(Constant)	-91.417	49.354		-1.852	0.070
Me-Go-H (°)	0.238	0.141	0.214	1.693	0.097
BMI (kg/m^2)	0.583	0.304	0.254	1.917	0.062
Sex	-6.803	2.769	-0.335	-2.457	.018*
ANB (°)	-0.039	0.406	-0.013	-0.096	0.924
Sum of Björk polygon angles (°)	0.229	0.132	0.227	1.730	0.090
R^2	0.392				
adjusted R^2	0.326				
F (df = 4; 54)	5.937				<0.001***
**6**					
(Constant)	-105.895	49.967		-2.119	0.039*
aC3-H-RGn (mm^2)	0.098	0.236	0.061	0.415	0.680
BMI (kg/m^2)	0.669	0.308	0.291	2.170	0.035*
Sex	-7.130	3.293	-0.351	-2.165	0.036*
ANB (°)	0.028	0.416	0.009	0.067	0.947
Sum of Björk polygon angles (°)	0.276	0.133	0.274	2.072	0.044*
R^2	0.357				
adjusted R^2	0.287				
F (df = 4; 54)	5.102				<0.001***

B unstandardized coefficient, *ß* standardized coefficient; * p < 0.05, ** p < 0.01, *** p < 0.001.
